# Supplementary material for: Deciphering the heterogeneity of the Lyve1+ perivascular macrophages in the mouse brain
Source: Nat Commun. 2022 Nov 30;13:7366. doi: 10.1038/s41467-022-35166-9 (PMC9712536; doi:10.1038/s41467-022-35166-9)
Supplement: Supplementary file 1 — Supplementary Information [file 41467_2022_35166_MOESM1_ESM.pdf]

Supplementary Information

Deciphering the heterogeneity of the Lyve1$^+$ perivascular Macrophages in the mouse brain

C. Siret, M. van Lessen, J. Bavais, H. W. Jeong, S. K. Reddy Samawar, K. Kapupara, S. Wang, M. Simic, L. de Fabritus, A. Tchoghandjian, M. Fallet, H. Huang, S. Sarrazin, M.H. Sieweke, R. Stumm, L. Sorokin, R. H. Adams, S. Schulte-Merker, F. Kiefer, S.A. van de Pavert

Supplementary Figures and Figure Legends 1-6

Supplementary Tables 1 and 2

Supplementary Figure 1

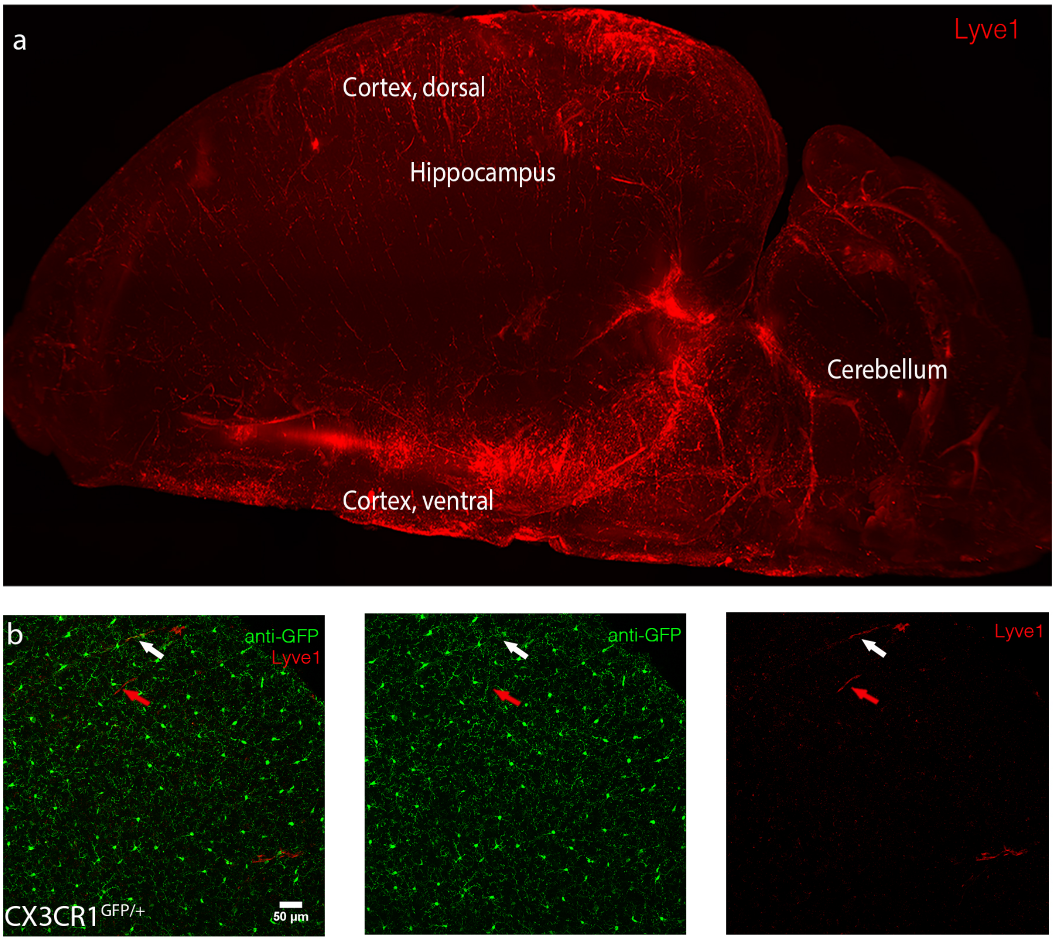

Supplementary Figure 1: CX3CR1⁻pvM characterization. (a) Lightsheet imaging of a cleared adult C57BL/6J mouse brain immunolabeled with anti-Lyve1 using the iDisco+ protocol (5550µm maximum intensity projection), related to video 1. (n=5) **(b)** Immunofluorescence microscopy on brain parenchyma sections of *Cx3cr1*^{GFP} in the dorsal cortex, using an anti-GFP to enhance the GFP signal (green) with Lyve1 staining labeled in red. White arrows show the conventional pvM population and red arrows show the non-conventional pvM population (n= 3).

Supplementary Figure 2

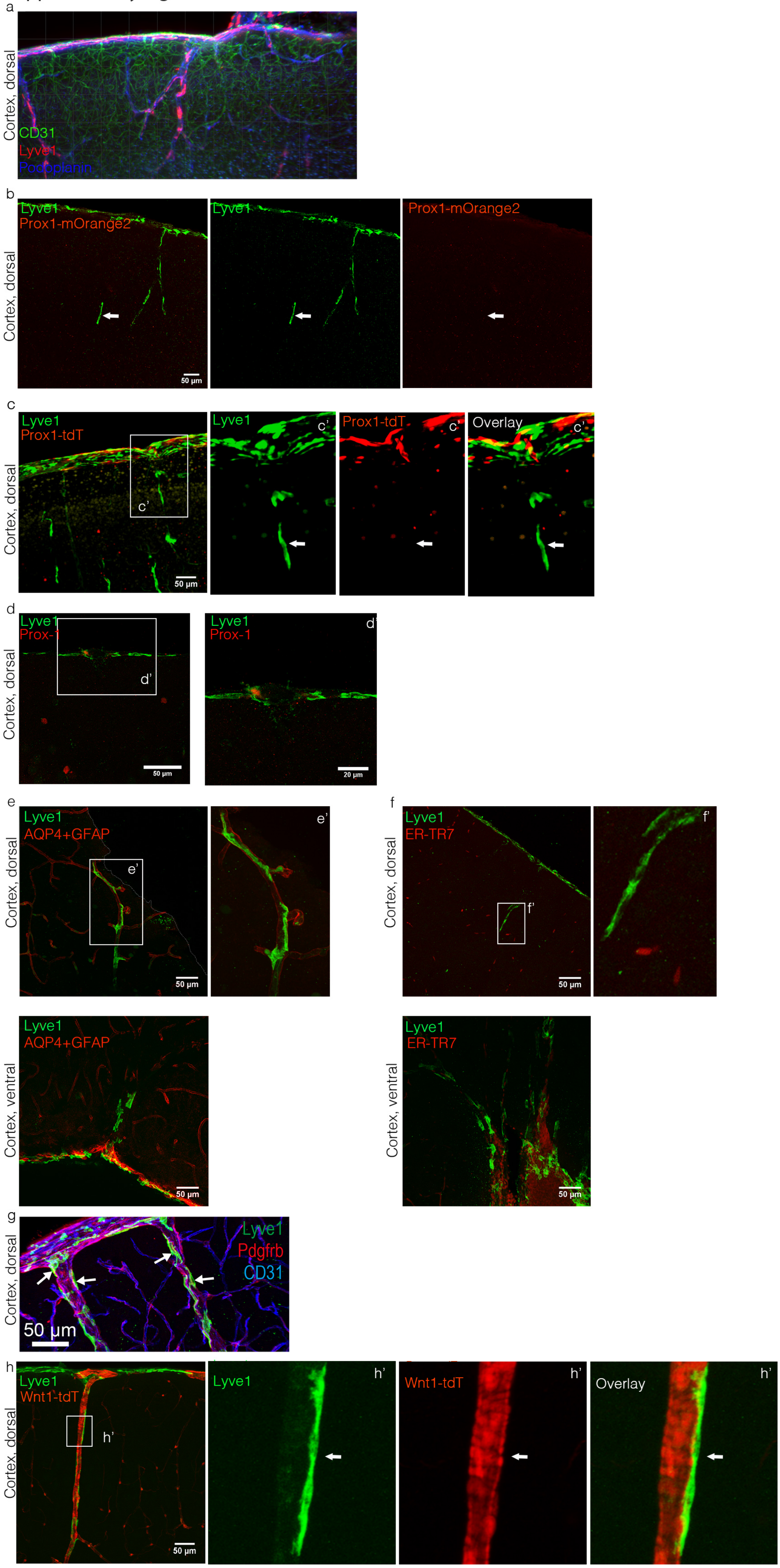

Supplementary Figure 2: Lyve1$^+$ cells are neither lymphatic endothelial cells (LEC), astrocytes, fibroblasts or pericytes. (a) 1080µm maximum intensity projection of lightsheet microscope acquisition of the dorsal cortex illustrating the « cul de sac of the pia mater » defined by the podoplanin labeling, structure emanating from the pia mater (CD31 in green, Lyve1 in red and podoplanin in blue) (n= 2). **(b)** Confocal analysis of *Prox*^{mOrange2} brain sections stained for Lyve1 (green) confirmed that Lyve1$^+$ cells do not express mOrange2 (red) (white arrows) (34µm maximum intensity projection). (n=4) **(c)** Confocal analysis of *Prox1-CreERT2*$^{+/-}$; *Rosa*^{tdTomato} brain sections stained for Lyve1 (green) confirmed that Lyve1$^+$ cells do not express tdTomato (red) (white arrows) (88µm maximum intensity projection) (n=2). **(d)** LEC identification (Lyve1$^+$ Prox1$^+$) in the pia mater in the dorsal cortex by Prox1 antibody staining (red) on C57BL/6J mouse brain in combination with Lyve1 (green). Prox1$^+$ neuron cellular bodies are observed in the parenchyma (13µm maximum intensity projection) (n=3). **(e)** Astrocyte identity was excluded by AQP4 and GFAP (red) staining (24µm maximum intensity projection for the dorsal cortex and 17µm maximum intensity projection for the ventral cortex) (n=3), **(f)** fibroblast identity was excluded by ER-TR7 staining (50µm maximum intensity projection for the dorsal cortex and 8µm maximum intensity projection for the ventral cortex) (n=3) and **(g)** pericyte identity was excluded by PDGFRβ (red) staining (n=1). **(h)** Confocal analysis of *Wnt-Cre; Rosa26*^{tdTomato} (37µm maximum intensity projection) brain sections stained for Lyve1 (green), containing tdTomato (red). (n= 3)

Supplementary Figure 3

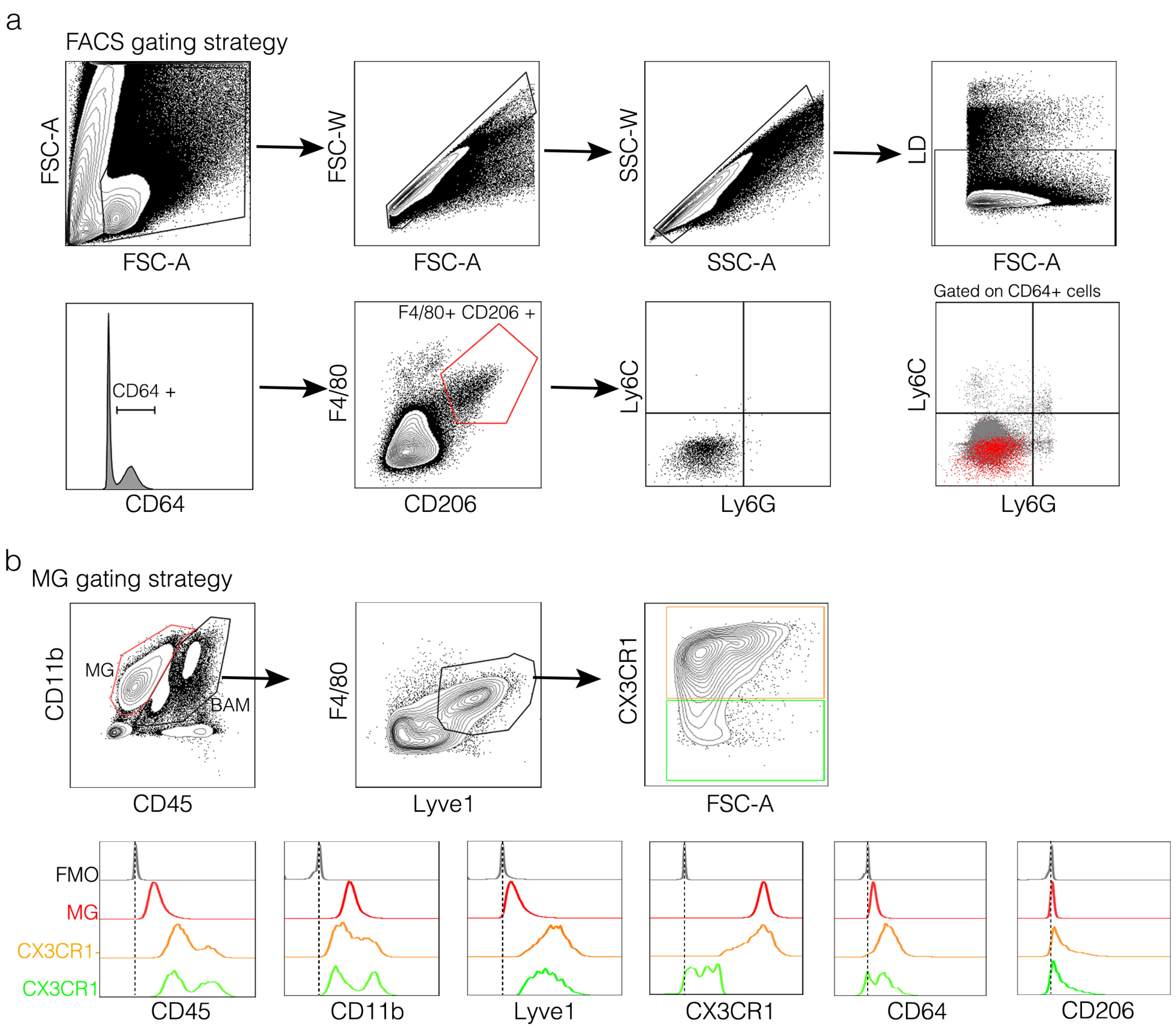

Supplementary Figure 3: Confirmation of the Lyve1^{+}CX3CR1^{-}population by flow cytometry (a) Flow cytometry gating strategy showing CD64^{+}F4/80^{+}CD206^{+}negative for Ly6C/Ly6C. **(b)** Gating of microglia (MG) and border-associated macrophages (BAM) isolated from *Cx3cr1^{GFP}* brain parenchyma and subsequent showing Lyve1^{+}F4/80^{+} BAM and CX3CR1^{+}/CX3CR1^{-}gating for these Lyve1^{+}F4/80^{+} BAMs and on histograms the expressions of CD45, CD11b, Lyve1, CX3CR1, CD64 and CD163 (in red for MG, in orange for Lyve1^{+} F4/80^{+}CX3CR1^{+} BAM and in green for Lyve1^{+}F4/80^{+}CX3CR1^{-}BAMs). FMOs are in grey. (1 individual experiment, n= 3 animals)

Supplementary Figure 4

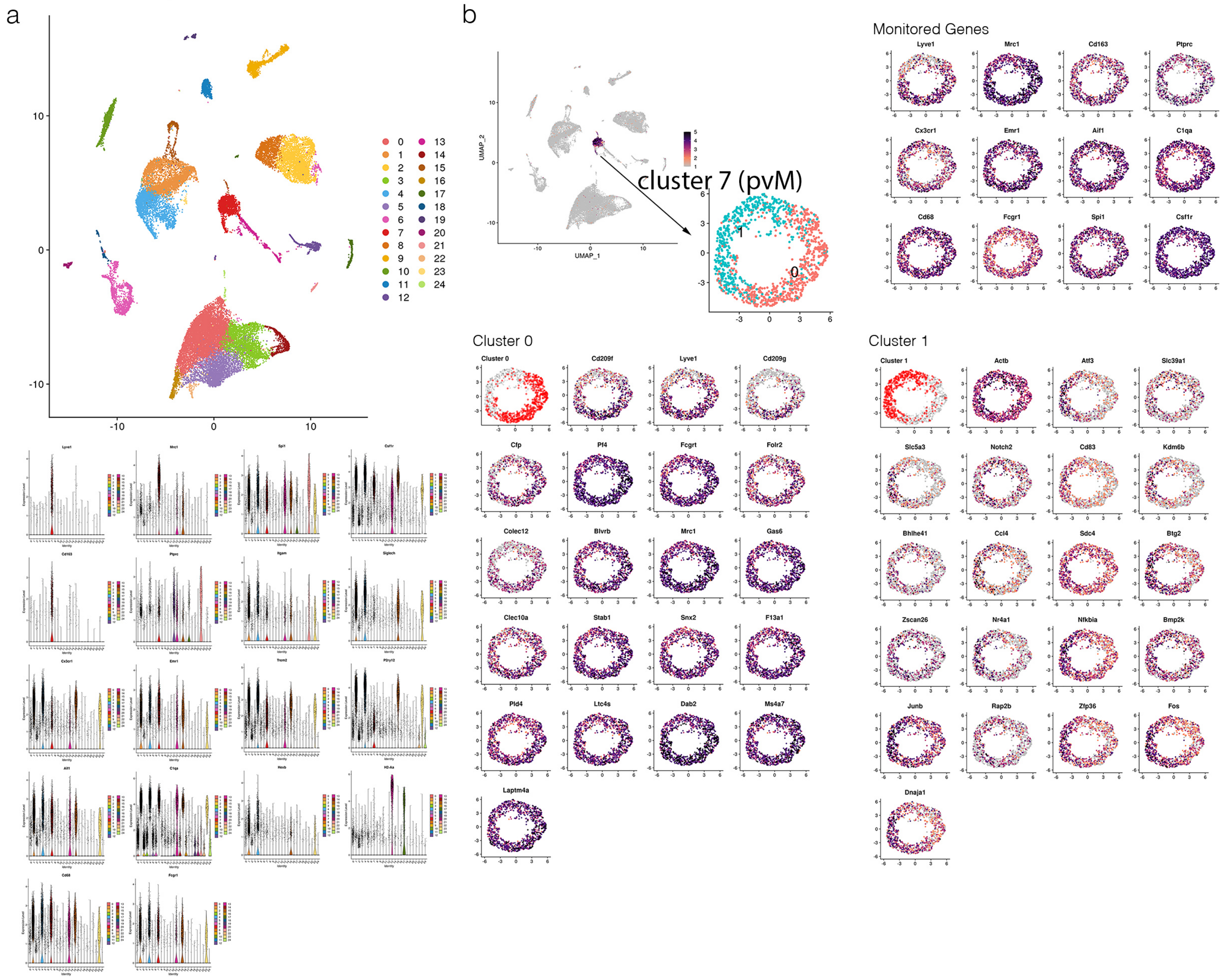

Supplementary Figure 4: Transcriptional profiling of the pvM subsets. (a) UMAP plot showing all the clusters. One cluster, number 7, contained *Lyve1*$^{+}$ expression as visualized in the Violin plot below. Selected genes associated with macrophages or microglia are further shown in the violin plots to determine exactly in which clusters they are expressed. **(b)** Cells from cluster 7 could be further segregated into 2 clusters. Segregation occurred on *Lyve1*, *Cd209f* and *Cd209g* and other genes show in the panel of cluster 0. In cluster 1, Fos pathway associated genes such as *Fos*, *Junb* and *Atf3* were higher expressed compared to cluster 0. However, as shown in the panel with the monitored genes, there was no segregation on *Cx3cr1*, *Ptprc* (encoding CD45) or *Spi1* (encoding PU.1).

Supplementary Figure 5

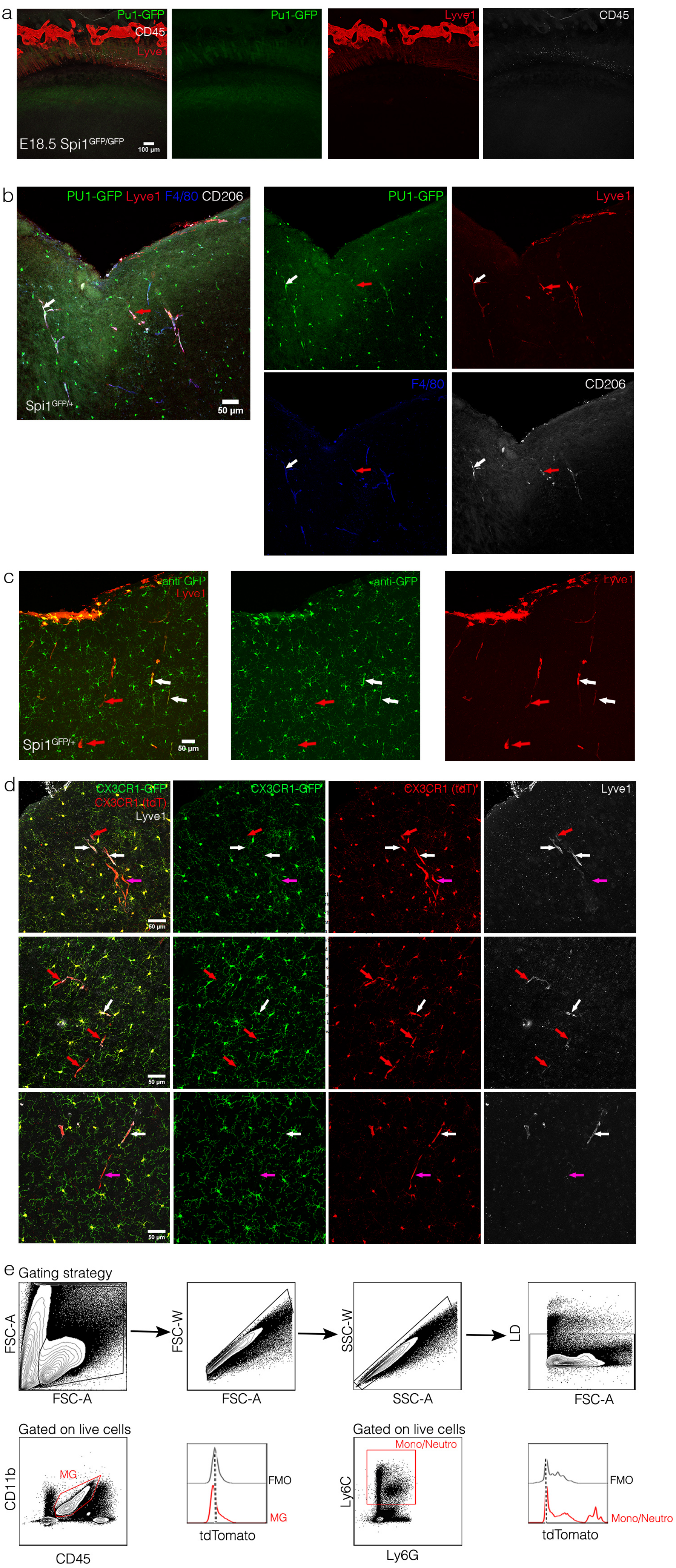

Supplementary Figure 5: The origin of CX3CR1⁻Lyve1⁺ perivascular macrophages. (a) Immunofluorescence of the section of a E18.5 head from a *Spi1*$^{GFP/+}$ embryo (PU1-GFP in green), labeled with αLyve1 (red) and αCD45 (white). No GFP or Lyve1 fluorescence was observed in the *Spi1*$^{GFP/GFP}$ E18.5 dorsal cortex (33µm maximum intensity projection) but we observed Lyve1$^+$ cells in the skull. **(b)** Confocal analysis of *Spi1*$^{GFP/+}$ in the dorsal cortex (25µm maximum intensity projection) brain sections stained for Lyve1 (red), F4/80 (blue) and CD206 (white). White arrows indicate the conventional pvM population and red arrows show the non-conventional pvM population. **(c)** Immunofluorescence microscopy on brain parenchyma sections of *Spi1*$^{GFP/+}$ in the dorsal cortex, using an anti-GFP to enhance the GFP signal (green) with Lyve1 staining labeled in red. White arrows indicate the conventional pvM population and red arrows show the non-conventional pvM population. **(d)** Different representative immunofluorescence stainings of adult *Cx3cr1*$^{GFP/+}$; *Cx3cr1-Cre*; *Rosa26*^{tdT} mouse brain sections, showing Lyve1$^+$CX3CR1$^+$ (white arrows), Lyve1$^+$CX3CR1$^-$ (red arrows) and Lyve1$^-$ pvM population (pink arrows) expressed tdTomato. (n= 3). **(e)** Flow cytometry gating strategy of *Cxcr4-CreErt2*; *Rosa26*^{tdT} brain parenchyma showing microglia (MG) lacking tdTomato reporter expression and monocytes/neutrophils (Mono/Neutro) partially positive for tdTomato.

Supplemental Figure 6

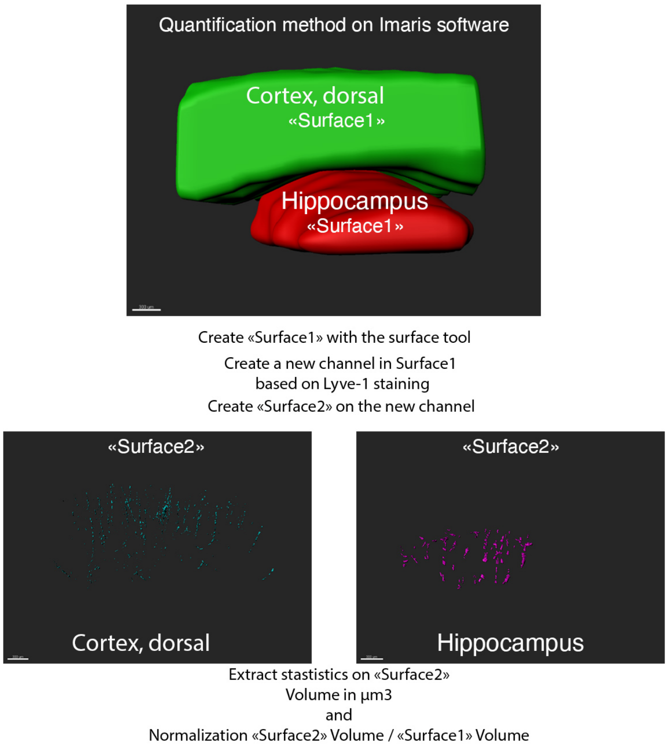

Supplementary Figure 6: Lightsheet data quantification method using Imaris software. To quantify the Lyve1 staining in cleared brains imaged by lightsheet microscopy, Imaris software was used. Using the "Surface tool" of Imaris, a 3D Rendering function, a first volume was created, named « Surface1 » at different locations (Dorsal cortex and hippocampus) where we wanted to quantify the staining. Based on Lyve1 labelling, a new channel was created. This new channel was used to create a new volume called « Surface 2 » representing the Lyve1 staining. Imaris calculated the different volumes in μm^3. The ratio « Surface2 » / « Surface1 » allows us to normalize the Lyve1 quantification.

Supplementary Table 1: Antibodies used for immunofluorescence and flow cytometry

Primary Antibodies	Source	Clone	Dilution	Identifier
Lyve-1	R&D systems	ND	1/500	AF2125
AQP4	EMD Millipore	ND	1/800	AB3594
CD45	eBioscience	30F11	1/250	14-0451-85
Iba1	Abcam	ND	1/500	ab107159
CD31	Thermo, abcam	ERMP12	1/200	MA1-40074, ab119341
Prox-1	Reliatech	ND	1/150	102-PA32
VEGFR3	R&D systems	ND	1/200	AF743
PDGFRβ	Cell Signaling	ND	1/200	3169S
ER-T7R	Thermofisher	ER-TR7	1/50	MA1-40076
GFP	AVES	ND	1/2000	GFP-1020
Podoplanin	Millipore	8.1.1	1/100	MABT-1512
Laminin α1	Sorokin L (University of Muenster)		n/a	317
Laminin γ1	Sorokin L (University of Muenster)		n/a	3E10

Primary Coupled Antibodies	Source	Dilution	Clone	Identifier
CD45-BUV395	BD Biosciences	1/300	30F11	564279
F4/80-BV421	Biolegend	1/200	BM8	123131
CD64-BV711	Biolegend	1/100	X54-5/7.1	139311
CD206-BV785	Biolegend	1/200	C068C2	141729
Ly6C-BV510	Biolegend	1/200	HK1.4	128033
Ly6G-AF700	BD Pharmingen	1/600	1A8	561236
Lyve1-eF660	eBioscience	1/50	ALY7	50-0443-82
CD163-SB600	eBioscience	1/200	TNKUPJ	63-1631-82
CD11b-BV605	BD Biosciences	1/200	M1/70	563015
GFAP-A647	Biolegend	1/200	2E1.E9	644706
MHCII-PE	Biolegend	1/400	M5/114	562352
F4/80-A647	Biolegend	1/200	BM8	123122
α-SMA-A488	Thermofisher	1/500	1A4	53-9760-82
**Viability marker**				
Zombie NIR Fixable Kit	Biolegend	1/500		423105

Secondary Antibodies	Source	Dilution	Identifier
DaChCy3	Jackson ImmunoResearch	1/400	703-166-155
DaG 488	Thermofisher	1/400	A-11055
DaG 555	Thermofisher	1/400	A-21432
DaG 647	Thermofisher	1/400	A-21447
DaG 790	Jackson ImmunoResearch	1/400	712-655-153
DaR 488	Thermofisher	1/400	A-21208
DaR 594	Thermofisher	1/400	SA5-10028
DaR 647	Jackson ImmunoResearch	1/400	712-605-153
DaRb 488	Thermofisher	1/400	A-21206
DaRb 555	Thermofisher	1/400	A-31572
DaRb 647	Jackson ImmunoResearch	1/400	711-605-152

Supplementary Table 2: Parameters used to filter cells in NGS analysis.
Cells were filtered out based on number of detected genes, number of UMIs and percentage of mitochondrial and ribosomal genes. Values adapted to each sample were used for this quality control step.

	Feature_max	Feature_min	Mitopct_max	Ribopct_min	UMI_max	UMI_min
Juvenile-1	4000	500	10	1	15000	500
Juvenile-2	5000	500	10	1	20000	500
Juvenile-3	5000	500	8	1	15000	500
Juvenile-4	4000	500	10	1	15000	500
Adult-1	4000	0	8	1	25000	500
Adult-2	4000	0	5	1	15000	500
Aged-1	3500	0	10	1	15000	500
Aged-2	3500	0	10	1	15000	500
