# Supplementary material for: Deciphering the heterogeneity of the Lyve1+ perivascular macrophages in the mouse brain
Source: Nat Commun. 2022 Nov 30;13:7366. doi: 10.1038/s41467-022-35166-9 (PMC9712536; doi:10.1038/s41467-022-35166-9)
Supplement: Supplementary file 3 — Description of Additional Supplementary Files [file 41467_2022_35166_MOESM3_ESM.pdf]

**Supplementary Movie 1: Lightsheet acquisition of a cleared sagittal half of an adult C57BL/6J mouse brain** immunolabeled with αLyve1 (red) on a blue background for orientation. The Movie zooms in on the ventral cortex and subsequently on the hippocampus to indicate the differences in pvM morphology within these regions.

**Supplementary Movie 2: Z-stack acquisition by confocal microscopy and 3D reconstruction of the pvM morphology adjacent to a blood vessel within the brain parenchyma.** A 100µm brain section was stained with αLyve1 (red) and αCD31 (green). 3D reconstruction was done using the Imaris surface tool and the Movie was created using the animation function of Imaris.

**Supplementary Movie 3: A CX3CR1 negative pvM.** 3D reconstruction of the CLSM acquired pvMs lining the blood vessel from Fig. 5g'. The irregular shaped pvMs (Lyve1, white) contained a nucleus (DAPI, yellow).

**Supplementary Movie 4: A CX3CR1 positive pvM.** 3D reconstruction of the CLSM acquired pvMs lining the blood vessel from Fig. 5g''. The irregular shaped pvMs contained a nucleus (DAPI, yellow).

**Supplementary Movie 5: Whole-mount acquisition and 3D rendering of the control brain for photothrombotic stroke.** Immunofluorescence of Lyve1 (red) in the whole brain. Movie of the area where the light was positioned, situated above the hippocampus. The invagination of the pia can be seen on the right, which extends to the 3$^{rd}$ ventricle (not shown). Lyve1$^{+}$ macrophages can be seen within the pia mater on top of the parenchyma and within the invagination. The Lyve1$^{+}$ pvMs are sparsely situated within the parenchyma.

**Supplementary Movie 6: Whole-mount acquisition and 3D rendering of the lesion in the photothrombotic stroke brain.** Immunofluorescence of Lyve1 (red) in the whole brain. Movie of the lesion area, situated above the hippocampus. The invagination of the pia can be seen on the right, which extends to the 3$^{rd}$ ventricle (not shown). Lyve1$^{+}$ macrophages have disappeared from the pia mater on top of the parenchyma but not within the invagination. Increased Lyve1$^{+}$ pvMs are situated within the parenchyma and in the hippocampus.
