# Supplementary material for: Deciphering the heterogeneity of the Lyve1+ perivascular macrophages in the mouse brain
Source: Nat Commun. 2022 Nov 30;13:7366. doi: 10.1038/s41467-022-35166-9 (PMC9712536; doi:10.1038/s41467-022-35166-9)
Supplement: Supplementary file 10 — Reporting Summary [file 41467_2022_35166_MOESM10_ESM.pdf]

nature research

Corresponding author(s): van de Pavert, Serge

Last updated by author(s): Nov 11, 2022

Reporting Summary

Nature Research wishes to improve the reproducibility of the work that we publish. This form provides structure for consistency and transparency in reporting. For further information on Nature Research policies, see our Editorial Policies and the Editorial Policy Checklist.

Statistics

For all statistical analyses, confirm that the following items are present in the figure legend, table legend, main text, or Methods section.

n/a	Confirmed	
☐	☒	The exact sample size (*n*) for each experimental group/condition, given as a discrete number and unit of measurement
☒	☐	A statement on whether measurements were taken from distinct samples or whether the same sample was measured repeatedly
☐	☒	The statistical test(s) used AND whether they are one- or two-sided<br>*Only common tests should be described solely by name; describe more complex techniques in the Methods section.*
☒	☐	A description of all covariates tested
☐	☒	A description of any assumptions or corrections, such as tests of normality and adjustment for multiple comparisons
☐	☒	A full description of the statistical parameters including central tendency (e.g. means) or other basic estimates (e.g. regression coefficient) AND variation (e.g. standard deviation) or associated estimates of uncertainty (e.g. confidence intervals)
☐	☒	For null hypothesis testing, the test statistic (e.g. *F*, *t*, *r*) with confidence intervals, effect sizes, degrees of freedom and *P* value noted<br>*Give P values as exact values whenever suitable.*
☒	☐	For Bayesian analysis, information on the choice of priors and Markov chain Monte Carlo settings
☒	☐	For hierarchical and complex designs, identification of the appropriate level for tests and full reporting of outcomes
☒	☐	Estimates of effect sizes (e.g. Cohen's *d*, Pearson's *r*), indicating how they were calculated

Our web collection on statistics for biologists contains articles on many of the points above.

Software and code

Policy information about availability of computer code

Data collection	FACS ARIA III SORP sorter, LSRFortessa X-20 cytometer (BD Biosiences), Diva software (BD Biosciences)<br>Lightsheet microscope Ultramicroscope II (LaVision BioTec, Miltenyi), Imspector software (version 5.1.304)(LaVision BioTec, Miltenyi)<br>Confocal microscope LSM880 (Zeiss), Zen blue (Zeiss)<br>10X Chromium system (10X Genomics), Illumina NextSeq 500 (Illumina), Cellranger (10x genomics)
Data analysis	FlowJo software (version 10) , Imaris software (version 9.1.0, Bitplane), ImageJ software (National Institutes of Health), GraphPad Prism (version 8.3.0), Seurat R package (version 4.1.0)

For manuscripts utilizing custom algorithms or software that are central to the research but not yet described in published literature, software must be made available to editors and reviewers. We strongly encourage code deposition in a community repository (e.g. GitHub). See the Nature Research guidelines for submitting code & software for further information.

Data

Policy information about availability of data

All manuscripts must include a data availability statement. This statement should provide the following information, where applicable:

- Accession codes, unique identifiers, or web links for publicly available datasets
- A list of figures that have associated raw data
- A description of any restrictions on data availability

The sequencing data generated in this study have been deposited in the GEO repository with the accession number GSE133283. Scripts and detailed instructions to reproduce the NGS analysis for this manuscript are available on GitHub repository https://github.com/JulieBvs/SPlab_PVM2

Field-specific reporting

Please select the one below that is the best fit for your research. If you are not sure, read the appropriate sections before making your selection.

[x] Life sciences [ ] Behavioural & social sciences [ ] Ecological, evolutionary & environmental sciences

For a reference copy of the document with all sections, see nature.com/documents/nr-reporting-summary-flat.pdf

Life sciences study design

All studies must disclose on these points even when the disclosure is negative.

Sample size	Power analysis was performed to estimatee the number of experimental mice
Data exclusions	No data exclusions unless stated otherwise
Replication	The experimental findings were reliably reproduced. We futher provide thoroughly detailed methods describing the critical staps of each experiment.
Randomization	No randomization was performed since animals were compared with co-housed litter-mate controls.
Blinding	N/A

Reporting for specific materials, systems and methods

We require information from authors about some types of materials, experimental systems and methods used in many studies. Here, indicate whether each material, system or method listed is relevant to your study. If you are not sure if a list item applies to your research, read the appropriate section before selecting a response.

Materials & experimental systems

n/a	Involved in the study	
[ ]	[x]	Antibodies
[x]	[ ]	Eukaryotic cell lines
[x]	[ ]	Palaeontology and archaeology
[ ]	[x]	Animals and other organisms
[x]	[ ]	Human research participants
[x]	[ ]	Clinical data
[x]	[ ]	Dual use research of concern

Methods

n/a	Involved in the study	
[x]	[ ]	ChIP-seq
[ ]	[x]	Flow cytometry
[x]	[ ]	MRI-based neuroimaging

Antibodies

Antibodies used	Antibodies used for microscopy:<br>Lyve-1 R&D systems (AF2125); AQP4 R&D systems (AB3594); GFAP Biolegend (644706); CD45 eBioscience (14-0451-85); F4/80 Biolegend (123122); Iba1 Abcam (ab107159); CD163 (Anders homemade); CD11b eBioscience (16-0112-82); CD206 Thermofisher (48-2061-82); CD31 Thermo (MA1-40074); Prox-1 Reliatech (102-PA32); VEGFR3 R&D systems (AF743); α-SMA Thermofisher (53-9760-82); PDGFRβ Cell Signaling (3169S); ER-T7R Thermofisher (MA1-40076); GFP AVES (GFP-1020); Laminin α1 Sorokin L (University of Muenster); Laminin γ1 Sorokin L (University of Muenster); DaChCy3 Jackson ImmunoResearch (703-166-155); DaG 488 Thermofisher (A-11055); DaG 555 Thermofisher (A-21432); DaG 647 Thermofisher A-21447; DaG 790 Jackson ImmunoResearch (712-655-153); DaR 488 Thermofisher (A-21208); DaR 594 Thermofisher (SA5-10028); DaR 647 Jackson ImmunoResearch (712-605-153); DaRb 488 Thermofisher (A-21206); DaRb 555 Thermofisher (A-31572);DaRb 647 Jackson ImmunoResearch (711-605-152)<br>Antibodies used for cytometry:<br>CD45-BUV395 (BD biosciences 564279), Lyve1-eFluor660 (eBioscience 50-0443-82) and F4/80-BV421 (Biolegend 123131)<br>CD64-BV711 (Biolegend 139311), CD206-BV785 (Biolegend 141729) Csf1R-PECy7 (Biolegend 135524), CD163-SB600 (eBioscience 63-1631-82), CD11b-BV605 (BD Biosciences 563015), MHCII-PE (Biolegend 562352), CD11b-BV605 (BD Biosciences 563015), CX3CR1-PECy7 (Biolegend 149015), Ly6C-BV510 (Biolegend 128033), LY6G-AF700 (BD Pharmingen 561236)
Validation	All the antibodies used in this studywere optimized and validated (i.e. assay and species) by suppliers and used according to supplied instructions. Most antibodies were re-evaluated and appropriate dilutions were determined by titration.

Animals and other organisms

Policy information about studies involving animals; ARRIVE guidelines recommended for reporting animal research

Laboratory animals	C57BL/6J mice were obtained from Charles River [France]. Prox-CreERT2+/- and Wnt1-Cre mice were kindly provided by Dr. Bajénoff (CIML, Marseille, France). Cx3cr1GFP mice were kindly provided by Dr. Lelouard (CIML, Marseille, France). Spi1GFP (PU.1) , Cxcr4-CreErt2;Rosa26tdT and Prox1mOrange2 were bred and maintained at the CIML (Marseille, France) under specific pathogen free conditions. Prox-CreERT2+/-, Wnt1-Cre and Cx3cr1-Cre lines were crossed to homozygosity for the tdTomato reporter using Rosa26tdT mice. P2, P7, P14, P21 and adult (8-10 weeks) and one year old mice (male and female) were used in this study. All mice were co-housed in the same room under similar conditions with water and food ad libitum and 12h/12h night/daylight cycle.
Wild animals	The study did not involve wild animals.
Field-collected samples	The study did not involve field-collected samples.
Ethics oversight	All experiments were reviewed and approved by the local ethics committee of Aix-Marseille University and the Ministère de l'Enseignement Supérieur, de la Recherche et de l'Innovation.

Note that full information on the approval of the study protocol must also be provided in the manuscript.

Flow Cytometry

Plots

Confirm that:

- [x] The axis labels state the marker and fluorochrome used (e.g. CD4-FITC).
- [x] The axis scales are clearly visible. Include numbers along axes only for bottom left plot of group (a 'group' is an analysis of identical markers).
- [x] All plots are contour plots with outliers or pseudocolor plots.
- [x] A numerical value for number of cells or percentage (with statistics) is provided.

Methodology

Sample preparation	Mice were anesthesized using Ketamine and Xylazine. Cx3cr1GFP mouse brains from male mice were collected after perfusion with ice cold PBS-Heparin (after removal of the dura). Brains were cut sagitally in 6-8 pieces, then digested with the Adult Brain Dissociation Kit (Miltenyi, 130-107-677) at 37°C for 30min on the gentleMACS dissociator (Miltenyi). Brain cell suspensions were filtered over 70µm strainers and filters were washed with 10mL HBSS 2%FBS. After centrifugation (5min, 400g, 4°C), debris were removed using a 40% Percoll (Sigma-Aldrich, GE17-0891-02) solution in PBS. Cells were centrifuged (30min, 500g, 4°C) and supernatant was discarded. Red blood cell lysis was performed for 10 min at 4°C using the Miltenyi RBC lysis buffer from the kit. Lysis was stopped with 9mL HBSS 2%FBS and cells were centrifuged (5min, 400g, 4°C). Cells were then blocked (15% normal mouse serum (Jackson Immunoresearch 015-000-120) in FACS buffer (HBSS 2% FBS) for 15 min and subsequently stained.
Instrument	FACS ARIA III SORP sorter, LSRFortessa X-20 cytometer (BD Biosiences)
Software	FlowJo software (version 10)
Cell population abundance	Sorted populations were >95%
Gating strategy	pvMs were defined as Lyve-1+F4/80+CD206+CD64+. Additional markers were employed to characterize pvMs: Cx3cr1, CD45, CD163.

- [x] Tick this box to confirm that a figure exemplifying the gating strategy is provided in the Supplementary Information.
